# 593 Development of a Burn Center Multi-Disciplinary Debriefing Process

**DOI:** 10.1093/jbcr/irac012.221

**Published:** 2022-03-23

**Authors:** Shannon Wyatt

**Affiliations:** Arkansas Children's, Benton, Arkansas

## Abstract

**Introduction:**

The purpose of debriefings are to help facilitate a healthier work environment for team members, improve processes, and improve patient outcomes. Debriefing staff members after specific events provides a time to support team member’s emotional needs, improve communication among the team members, and improve patient outcomes (Schulman, Jhanwar, and Shara, 2015; Wolfe et al., 2014). Multi-disciplinary team members working in Burn Units are repeatedly exposed to stressful patient events such as end of life, escalation of care, major burn admissions, or mass casualty events. These traumatic exposures can all increase the risk of staff burnout, which is negatively associated with resilience (Colville et al., 2017).

In 2019, our Burn Unit had 7 events that met criteria for a formal debrief but had no process for conducting these debriefs. Without a formal team debrief there is no loop closure of an event for staff and no platform to discuss potential ways to improve practice. The purpose of this project was to develop a formal debrief process and institute the process on the Burn Unit.

**Methods:**

Criteria for debriefing were identified as adults and pediatrics requiring CPR, end-of-life care, and emergent escalation of care. Six registered nurses volunteered to be Debrief Facilitators (DFs) and underwent training utilizing real-time simulation scenarios conducted by the Simulation Education Center. DFs were provided a Process Map along with debrief tools and resources needed to conduct a formal debrief. Facilitators were responsible for initiating, organizing, facilitating, and documenting a debrief after an event that met criteria.

**Results:**

The formal debrief process was initiated in September of 2020. Since the initiation, 12 events have met criteria for debrief. 12 formal debriefs were conducted during the following 10 months.

**Conclusions:**

After a formal debrief process was identified and education provided by the Simulation Education Center the Burn Unit conducted 100% of events that met criteria for debriefing through June of 2021. In conclusion, giving team members formal processes, tools, and educational opportunities has proven successful for formal debriefs in the Burn Unit.

